# 

**Published:** 1830-04-01

**Authors:** 


### LITERARY NOTICES.

In the press—" The Physiology of the Fœtus, the Liver, and the Spleen." By G. CALVERT HOLLAND.

A Gentleman proposes to publish a work, entitled " Analecta Medica, or Gleanings from the Portfolio of Iatros," which will comprise an analysis of the practical matter contained in the greater number of the *medical* works that have been published in this country during the last thirty or forty years. To this undertaking he has been prompted, as well by a persuasion that such a work is much wanted, by a large proportion of the profession, as by the circumstance of his possessing a very large mass of materials, occupying no less space than fifteen thick quartos, the labour of his own pen, and the result of the habit of analyzing, for his own private use, in the course of perusal, the works which have fallen in his way. From this source he proposes to select and arrange, under proper heads, the most useful portions, divested of every thing of a speculative character. The plan will not embrace, for obvious reasons, several of the more recent standard works on the practice of medicine, nor several of the late monographs, which ought to be in the hands of every respectable practitioner. He designs sending forth a common-sized octavo annually, complete, or in half-yearly parts. An Appendix will be added from time to time, containing an outline of the *cases* given by the authors cited, which being arranged, may be bound up in a separate volume, forming a useful " Clinical Case-book," that may be consulted either with, or without the rest of the work. As conciseness is an indispensable requisite in the execution of such a plan, the original matter will be compressed as much as possible, the ipsissima verba of his authors only being given on points of essential moment, where perspicuity and weight might possibly be otherwise sacrificed at the shrine of brevity and obscurity. The *phraseology* will be couched in the simple abbreviated style best adapted to the purposes of a note book. The main scope of the work, then, unlike that either of an elementary or systematic one, or of a mere catalogue raisonné, is to give simply a succinct, digested view of much important pathologic and therapeutic matter, which lies widely scattered through some hundred volumes, the utility of which is for the most part, on that account, unavailable to the busy practitioner at the juncture when he most particularly requires it. It is, therefore, presumed that these volumes will form a work of general reference and consultation of no mean value. But the public reception of the first volume or two will be the measure of the compiler's future labours in this ample field. In his original analysis, the diseases were grouped *anatomically*, according to their seats, merely for the sake of convenience, and as this is a point of little importance while a table of contents is given at the commencement, and a special index at the end, of the volumes, this arrangement will be still pursued, thus:
α Diseases of Brain and Nervous System—β Of Respiratory Organs—γ Of Sanguiferous System (Fever, Inflammation, &c.)—δ Of Chylopoietic Viscera—ε Of Absorbent and Glandular System—ζ Of Organs of Sense—η Of the Muscular System—ϑ Of the common Integuments—ι Of the Urinary Organs—κ Of the Organs of Generation—λ Of the Bones and Appendages.

A catalogue of the preparations in the *Anatomical Museum of Guy's Hospital* arranged and edited by desire of the Treasurer of the Hospital, and of the Teachers of the Medical and Surgical School, by Dr. Hodgkin, Demonstrator of Morbid Anatomy and Curator of the Museum of Guy's Hospital, is just published by Mr. Highley.

Sir ASTLEY COOPER has just brought out a splendid work on the Structure and Diseases of the Testis, accompanied by 24 beautiful coloured plates.

Mr. CURTIS, Surgeon Aurist to His Majesty, has just published a Clinical Report of the Royal Dispensary for Diseases of the Ear, from 1816 to 1830, containing an account of the number of patients admitted, cured, and relieved, illustrated with cases and practical remarks relative to the various affections of the ear.

### *New Medical Journal in Ireland.*

We have received the prospectus of a new monthly medical journal to be published in Clonmel, under the editorial direction of Mr. D. Philan. It is an arduous undertaking; but we sincerely wish it success.

### BIBLIOGRAPHICAL RECORD;

OR,

*Works received for Review from the 25th January to the 25th March, 1830.*

1. Researches principally relative to the Morbid and Curative Effects of Loss of Blood. By MARSHALL HALL, M.D. Octavo, pp. 302. London, 1830.

---

2. A Treatise on Fever. By SOUTHWOOD SMITH, M.D. Physician to the London Fever Hospital. Octavo, pp. 436. London, 1830.

---

3. A new Supplement to the Pharmacopœias of London, Edinburgh, Dublin and Paris; forming a complete Dispensatory and Conspectus, including the new French Medicines and Poisons; with Symptoms, Treatment, and Tests, &c. By J. RENNIE, A.M. A.L.S., &c. Octavo, pp. 488. London, 1829.

☞ *This edition is revised and considerably enlarged—and thus rendered much more useful.*

---

4. A Treatise on Poisons, in relation to Medical Jurisprudence, Physiology, and the Practice of Physic. By R. CHRISTISON, M.D. Professor of Medical Jurisprudence and Police in the University of Edinburgh, &c. Octavo, pp. 698, 1 Plate. Edinburgh, 1829.

---

5. A Letter to the Public on the Necessity of Anatomical Pursuits; with reference to popular Prejudices, and to the Principles on which Legislative Interference in these Matters ought to proceed. By CORDEN THOMPSON, M.D. Octavo, stitched, pp. 92. London, 1830.

---

6. Practical Remarks on Amputations, Fractures, and Strictures of the Urethra. By STEPHEN LOVE HAMMICK, Surgeon Extraordinary to the King, &c. Octavo, pp. 265. London, 1830.

---

7. A Synoptical, Meteorological, and Symptomatological Journal, or Medical Case Book of Record; containing Prescription-Book and Ledger. By W. R. RUSSEL WILTON, Surgeon, &c. Price from 5s. 6d. to 31s. 6d. according to Size and Quantity of Sheets in each. Large post-folio, (quarto) and sexta, bound in Parchment. Bath, 1829.

---

8. Manual of Therapeutics. By L. MARTINET, D.M.P. Translated, with Alterations and Additions, by ROBERT NORTON, M.D. &c. 18mo. pp. 323. Jackson, London, 1830.

☞ *A very useful little book to students, to whom we recommend it.*

---

9. A new System of treating the Human Teeth, &c. By J. PATERSON CLARK, M.A. Dentist. Octavo, pp. 199, 2d Ed. Longman and Co. 1830.

---

10. An Exposure of the Causes of the present deteriorated Condition of Health, and diminished Duration of Human Life, compared with that which is attainable by Nature, &c. forming a Code of Health and long Life. By JOEL PINNEY, Esq. Octavo, pp. 323. Longman's and Co. 1830.

---

11. On the Art of preserving Health in India. By T. E. BAKER, Surgeon of the 10th Light Cavalry. Octavo, pp. 66. Calcutta, 1829.

---

12. Observations on the Functional Disorders of the Kidneys, which give rise to the Formation of Urinary Calculi; with Remarks on their Frequency in the County of Norfolk. By WILLIAM ENGLAND, M.D. Octavo, pp. 108. Underwood, 1830.

---

13. A popular Summary of Vaccination, with reference to its Efficacy and probable Causes of Failure, as suggested by extensive practical Experience. By JOHN MARSHALL, Esq. M.R.C.S. District Vaccinator to the National Vaccine Establishment. Octavo, pp. 95. London, 1830.

---

14. A. Corn. Celsi de Re Medica. Libri Octo. Editio nova ex recensione Leo Targa. Curante C. F. COLLIER, M.D.; accedit Lexicon Celsiarum breve. Vol. 2, 18mo. S. Highley, Londini, 1829, 4s.

☞ *A very convenient little edition of the excellent work of Cornelius Celsus. Those students who are qualifying for Apothecaries' Hall will appreciate the Work, no doubt.*

15. A Manual of the Economy of the Human Body in Health and Disease; containing a brief View of its Structure and Functions, and the Diseases to which it is liable; with ample Directions for the Regulation of Diet and Regimen from Infancy to old Age. For the Use of General Readers. Octavo, pp. 417. Edinburgh, 1830.

*A very well-written volume.*

16. Introductory Lectures to a Course of Military Surgery, delivered in the University of Edinburgh. By GEORGE BALLINGALL, M.D F.R.S. E. Regius Professor of Military Surgery, &c. Octavo, pp. 246. Edinburgh and London, 1830.

17. A practical Essay on Stricture of the Rectum; illustrated by Cases, &c. to which is now added some practical Observations on Piles and the Hæmorrhoidal Excrescence. By FREDERICK SALMON, Senior Surgeon to the General Dispensary, Aldersgate Street. 3d Ed. very materially enlarged. Octavo, pp. 272. London, 1829.

*An improved edition of a useful Work.*

18. Illustrations of some of the principal Diseases of the Ovaria, their Symptoms and Treatment; to which are prefixed, Observations on the Structure and Functions of these Parts in the Human Being and in Animals. By EDWARD J. SEYMOUR, M.D. Fellow of the Royal College of Physicians and one of the Physicians to St. George's Hospital. With 14 Lithographic Engravings; 8vo. (the letter-press), pp. 128. 1830.

19. A practical Formulary of the Parisian Hospitals, &c. Translated from the 3d Edition of M. Ratier. By R. D. M'LELLAN, M.D. Duodecimo, pp. 272. 1830.

20. A Treatise on the Nature and Causes of the Yellow Fever; with Observations on the Mediterranean Fevers, &c. By ALEX HEASTIE, Surgeon, R.N. Octavo, pp. 150. 1830.

21. Observations and Cases relative to Dislocations of the Shoulder-joint, with a Variety of Methods for Reduction, &c. By R. ROBERTS. Octavo, pp. 161, with several Lithographs. 1830.

*The author believes he has discovered a Method or Methods of reducing Dislocations of the Humerus, superior to any now in use. We have heard, also, that he has been very successful. But the work is insusceptible of analysis, and cannot be understood without the engravings. Our surgical brethren, therefore, must have recourse to the original—an* ORIGINAL *in every sense; for, although on the dull and painful subject of dislocations, Mr. Roberts' book kept us convulsed with laughter for an hour by the clock.*

22. Lectures on Practical and Medical Surgery, comprising Observations and Reflexions on Surgical Education; on the Investigation of Disease; and on the ordinary Duties of the Surgeon, forming part of an extended Course of the Prinples and Practice of Surgery, delivered in 1828-9, illustrated by Engravings. By THOMAS ALCOCK, M.R.C.S. &c. Octavo, pp. 302, with coloured Engravings: also in small 8vo, without Engravings.

23. On the Nature and Treatment of the most frequent Diseases of Children, with Observations on the Management of early Infancy, &c. By MILES MARLEY, F.L.S. Member of the Royal College of Surgeons. Octavo, pp. 312. 1830.

24. Hints for the Suppression and Extinction of Fires, &c. By ROBERT VENABLES, M.B. Sewed, pp. 16. Price 6d.

25. Proceedings of the Eleventh Anniversary Meeting of the Hunterian Society. 1830.

26. A Dissertation on the Influence of Heat and Humidity;—with practical Observations on the Inhalation of Iodine and various Vapours in Consumption, Catarrh, Croup, Asthma, and other Diseases. By JAMES MURRAY, M.D. &c. Octavo, pp. 305. 1830.

27. Observations on the Pathology of Venereal Affections. By BENJAMIN TRAVERS, Esq. F.R.S. and Senior Surgeon to St. Thomas's Hospital. Octavo, pp. 74. 1830.

28. A Vade Mecum of Morbid Anatomy, Medical and Chirurgical; with pathological Observations and Symptoms. Illustrated by upwards of 250 Drawings. Royal 8vo. Burgess and Hill, 1830, 25s.

29. A Treatise on Hysteria. By GEO. TATE, M.R.C.S. Octavo, pp. 134. 1830.

### INDEX.

A.

Abdomen, morbid appearances of, in fever .......... 358
Abdominal inflammation, Mr. Bates on .......... 313
Abdominal inflammation, treatment of .......... 314
Abdominal complication in fever .. 351
Abercrombie, Dr. on a peculiar delirium .......... 197
Abercrombie on a peculiar affection of the pericranium .......... 256
Abercrombie, Dr. on ramollissement of the cord .......... 487
Abernethian doctrines, criticisms on the .......... 296
Abscess around and in the kidney.. 82
Accumulations in the colon, treatment of .......... 142
Acetate of morphine and morphia.. 545
Action of the heart, Dr. Williams on the sounds of the .......... 155
Actual cautery, employment of the . 190
Ague, topical inflammations in .... 307
Air and exercise in phthisis pulmonalis .......... 241
Air and pure water, action of, on lead 491
Alibert, Baron, on the leuca of the ancients .......... 523
American indignation at English libellers .......... 455
Amputations, &c. Mr. Hammick on 405
Anatomie Pathologique, by M. Cruveilhier .......... 79
Anatomy, general, Mr. Grainger's.. 49
Anatomy, lectures on, by Mr. B. Cooper .......... 95
Anatomy, Dr. Thomson on the necessity of .......... 332
Anatomy of a Chinese foot .......... 494
Anatomy bill, Mr. Guthrie on the.. 573
Andral M. on phthisis .......... 97
Aneurism after venesetion .......... 476
Aneurism, remarks on .......... 502
Angina pectoris .......... 126
Animals, experiments on, in cases of poisoning .......... 373
Annesley, Mr. on accumulations in the colon .......... 138
Annesley, Mr. on displacement of the colon .......... 570
Anus, prolapsed, treated in Mr. Hey's manner .......... 154
Apoplexy, interesting cases of .... 204
Apoplexy simulated by gastric irritation .......... 251
Apoplexy, interesting cases of .... 457
Apoplexy of the lungs, curious cases of .......... 555
Apothecaries' Company, regulations of the .......... 169
Apprenticeship system, injustice of, 446
Armstrong, Dr. biographical sketch of .......... 281
Arnott, Mr. on inflammation of the veins .......... 1
Arnott, Dr. his elements of physics 334
Artificial anus opening into the vagina, case of .......... 151
Ascending paralysis, curious case of 161
Ascites, iodine in .......... 249
Asthma—enormous hypertrophy of the heart .......... 463

B.

Bacot, Mr. on syphilis .......... 548
Bally, M. on morphine .......... 545
Bardsley, Dr. his hospital facts, &c. 129
Barry, Dr. on the Gibraltar fever.. 539
Bates, Mr. on abdominal inflammation .......... 313
Baucal, M. his manual of Lithotrity 228
Biographical sketch of Dr. Armstrong .......... 281
Bleeding in fever .......... 389
Blistered surfaces treated by carded cotton .......... 572
Blood-letting in fever .......... 399
Blood-letting, Dr. Marshall Hall on 40
Blistering for ulcers .......... 474
Body, changes of, during existence, 54
Bostock, Dr. on Thames water .... 443
Bowels, extraordinary constipation, 181
Brain and spine, irritation of the .. 293
Brain, extensive disease of .......... 460
Brain, partial ramollisement of the 215
Bransby Cooper's description of a Chinese foot .......... 494
Breast, fungus hæmatodes of the.. 238
Bright, Dr. his report of medical cases .......... 97
Burns and scalds .......... 199
Burns, carded cotton for .......... 570
Burrows, Dr. and Dr. Gooch .......... 278

C.

Cæcum and colon, displacement of, 571
Cæsarian operation on a dead woman 521

Cameron, Mr. on nitre in scurvy .. 483
Cancer of the kidney, case of...... 81
Cancer treated by compression.... 490
Cancer mammæ, cases of........ 525
Cannula for tying the polypus uteri, plan of, &c.................... 328
Carded cotton for blistered surfaces, 572
Carditis, supposed fatal case of .... 149
Carotid aneurism (supposed) ligature ultrà tumorem ................ 165
"Carotid aneurism"............... 501
Casamayor, M. on recto-vaginal fistula........................ 151
Cataracts alternating with diabetes 176
Cerebral complication in fever .... 349
Cerebral disease, rapidly fatal case of 458
Cerebro-spinal irritation, dangers of the doctrine .................. 297
Cervix uteri, state of, in pregnancy 44
Chemical analysis in cases of poisoning ........................ 371
Children, Mr. Marley on the diseases of .................... 553
Chinese foot, anatomical description 494
Chlorosis, amenorrhœa, &c. cases of 186
Christison, Dr. his treatise on poisons ........................ 362
Christison, Dr on lead .......... 491
Chronic hepatitis, hysteria mistaken for ........................ 240
Clavicle, dislocation of, forwards .. 178
Climate, Dr. Clark on............ 90
College of Physicians, soirées of the 520
Colon, Mr. Annesley on disorders of, 138
Colon, stricture of the sigmoid flexure of the .................. 425
Colon, fatal stricture of the........ 504
Company of Apothecaries, regulations of the .................. 169
Congestion, pseudo, of the brain .. 75
Consolations in travel........... 401
Constipation of the bowels, extraordinary case of ................ 181
Consumption, Dr. Parrish on...... 241
Contracted mitral valve, diagnosis of......................... 561
Cooper, Mr. Bransby, his anatomy 95
Cord, encysted hydrocele of the.... 269
Cruveilhier, M. his anatomie pathologique .................... 79
Cupping-glasses applied in a case of viper-bite .................... 175
Curriculum of the Irish College.... 446
Cynanche trachealis, cases of...... 218
Cyst, ovarian, injection of the .... 427
Cystocele, long mistaken case of .. 444

D.

Davies, Mr. lunatic commission on 433
Davy's, Sir H., consolations in travel 401
Delirium, Dr. Abercrombie on .... 197
Delirium tremens, Dr. Weir on.... 568
Delpech, M. on elephantiasis ...... 535
Depletion in dissection-wounds.... 533
Diabetes, cataracts alternating with 176
Diagnosis of hernia .............. 450
Dilatation of the urethra in the female for stone ................ 209
Dilator vaginæ found in Pompeii .. 247
Dinner of the general practitioners 518
Disease, spread of................ 300
Dislocation of the cornua of the os hyoides ...................... 159
Dislocation of the clavicle ........ 178
Dislocation of the head of the radius backwards ................ 178
Dissection-wound, case of ........ 522
Dissection-wounds, Mr. Lawrence on 529
Distortion of the face cured by disease of the brain !.............. 264
Dorsal vertebræ, removal of ...... 207
Dropsy, encysted, of the ovaria.... 419
Dupuytren, M. his rhinoplastic operation ........................ 268
Dupuytren, M. on specks of the cornea ...................... 539
Dupuytren, M. his treatment of scrofula ...................... 554

E.

Earle, Mr. on vesico-vaginal fistula, 272
Egotism extraordinary............ 280
Elbow-joint, excision of the ...... 189
Elementary fibre and tissues........ 53
Elephantiasis of the scrotum ...... 505
Elephantiasis of the scrotum...... 535
Elongation and displacement of the colon ........................ 570
Emphysema of the lungs.......... 200
Empyema with external tumour.... 238
Empyema and pneumo-thorax .... 466
Encysted hydrocele of the cord .... 269
England, Dr. on the kidneys ...... 481
English medical politics .......... 454
Epilepsy cured by trephining...... 504
Ethics, modern medical .......... 145
Excision of nerves in tic douloureux, 471
Exercise, corporeal, benefits of.... 303
Exposure to air in phthisis ........ 241
Extirpation of the ovaria, cases of.. 440
Extravasation of urine, fatal case of, 275
Eye, destruction of, in parturition.. 35
Eye, fungus hæmatodes of the ... 236

F.

Face, distortion of the, oddly cured, 264
Female, lithotrity unsuccessful in the 209
Female breast, tumours of the .... 524
Femoral hernia, diagnosis of ...... 453
Fever, Dr. Smith on.............. 337

Fever, Dr. Tweedie on ........... 337
Fever, doctrines of .............. 338
Fever, essential symptoms of ...... 340
Fever, Dr. Johnson's views of...... 342
Fever and inflammation not the same ..................... 344
Fever, synochus, description of .. 346
Fever, functional disturbance in ... 347
Fever, Drs. Smith and Tweedie on, 385
Fever, causes of ................ 385
Fever, treatment of.............. 386
Fever, causes of ................ 396
Flour, the panacea for burns ...... 199
Fœtus, fungus hæmatodes in the .. 163
Fœtus in utero, vaccination of the.. 214
Foreign opinions on medical politics, 454
Formulary of the Parisian hospitals, 540
Fractures, successful issue of...... 172
Fractures, simple and compound .. 566
Fungoid disease of the ovaria...... 422
Fungus hæmatodes of the brain.... 217
Fungus hæmatodes, cases of ...... 235
Fungus hæmatodes of stomach and liver ........................ 563
Fungus of the dura mater ........ 489

G.

Gastric irritation ................ 251
General anatomy, Mr. Grainger's .. 49
General poisoning, remarks on .. 365
General practitioners ............ 486
General practitioners, dinner of the, 518
Gibbs, Dr. his removal of a nervous tumour ...................... 184
Gibraltar fever, Dr. Barry on the .. 539
Godman, Dr. on tight lacing ...... 512
Gonorrhœal rheumatism.......... 548
Gooch, Dr. on pregnancy.......... 42
Gooch, Dr. on pseudo-congestion of the brain in children............ 75
Gooch, Dr. on uterine hæmorrhage, 194
Gooch, Dr. on polypus uteri ...... 321
Guthrie, Mr. on oil of turpentine .. 232
Guthrie, Mr. his Hunterian oration, 528
Guthrie, Mr. on the anatomy bill .. 573

H.

Hæmorrhage, peculiar, from the uterus......................... 194
Hall, Dr. Marshall, on blood-letting, 40
Hammick, Mr. on Amputations, &c. 405
Head, morbid appearances of, in fever ......................... 357
Heart, apoplexy of the............ 88
Heart, neuralgia of the .......... 122
Heart, Dr. Williams on the auscultation of the .................. 155
Heart, state of, in sleep .......... 299
Heart, hypertrophy of right side of, 555
Hemiplegia—strychnine employed, 239
Hennen, Dr. his military surgery, 361
Hepatitis, hysteria mistaken for ... 240
Hernia, inguinal, new species of ... 180
Hernia of the bladder, case of .... 444
Hernia, on the diagnosis of ....... 459
Hernia, umbilical, remarkable case of.............................. 463
Hooping-cough, Dr. Palmer on .... 308
Hospital facts and observations, Dr. Bardsley's ................... 129
Hospital for children at Paris .... 541
Hospitals and infirmaries, advantages of ....................... 146
Hunterian oration, Mr. Guthrie's .. 528
Hydatid tumour of the breast ..... 527
Hydatids of the placenta, M. Cruveilhier on.................. 80
Hydrocele, Mr. Symes on ........ 192
Hypertrophy, enormous, of the heart 463
Hypochondriasis, remarkable case of 509
Hysteria, cases of and remarks on.. 186

I.

Iliac abscesses and tumours, M. Teallier on ...................... 160
Inflammation of the veins ........ 1
Inflammation of the veins, secondary affection in ............... 19
Inflammations simulated by hysteria 186
Inflammation of the spleen, cases of 252
Inflammation of the peritoneum .. 313
Inflammation and fever .......... 344
Inflammation of the ovarium...... 417
Inguinal hernia, new species of.... 180
Inguinal hernia, remarks on ...... 450
Injuries of the head.............. 28
Injury, fatal, of the knee-joint .... 230
Intellect and disease, march of .... 300
Intellectual labour, deplorable effects of ...................... 302
Intermittent ophthalmia.......... 534
Insanity—trial of Mr. Davies...... 433
Iodine in bronchocele, scrofula, and ascites ...................... 248
Iodide of mercury in lupus ........ 570
Iodine, morbid effects of.......... 572
Irish College of Surgeons, curriculum of the...................... 446
Iritis, oil of turpentine in ........ 232
Irritable breast, case of .......... 524
Irritation of the brain and spine, Dr. Palmer on ................ 293
Itching, morbid, of the scrotum ... 493

J.

Jackson, Dr. on cynanche ........ 218
Jadelot, M. on infantile diseases .. 541
Jahn, Dr. on the bad effects of iodine 572
Jewel, Mr. on nitrate of silver .. 517
Joints, affections of, after injuries.. 24

Joints, affections of............... 31
Joints, affection of............... 383

K.

Kidney, cancer and abscess of the.. 81
Kidneys, Dr. England on disorder of the........................ 481
Kings of France, post-mortem examinations of the............... 259
Knee-joint, fatal injury of the .... 230
Knee-joint, loose cartilages in the, 562

L.

Labia pudendi, elephantiasis of the, 508
Lacerated wound in the arm ...... 174
Lacing, tight, injurious effects of .. 512
Lavements, Mr. Scott on ......... 499
Lawrence, Mr on dissection-wounds 529
Lead, action of air and water on .. 490
Lee, Dr. on uterine phlebitis ...... 376
Leeches, fatal application of...... 76
Leuca, Baron Alibert on.......... 523
Leucorrhœa ..................... 196
Leucorrhœa, nitrate of silver in.... 517
Liberty of the press .............. 438
Ligature ultrà tumorem, for supposed carotid aneurism ........ 165
Ligature for polypus uteri ........ 326
Ligature ultrà tumorem ........ 501
Liquor potassæ in ovarian disease.. 431
Literary property and reclamations, 278
Lithotomy, Mr. Stanley on...... 69
Lithotomy, mode of performing ... 70
Lithotrity in the female .......... 209
Lithotrity and lithotomy.......... 227
Loose cartilages in the knee-joint, 562
Lower jaw, half removed ........ 469
Lower lip destroyed—rhino-plastic operation ..................... 268
Lunacy *versus* sanity ............ 433
Lungs, state of, in phthisis ...... 98
Lungs, emphysema of the ........ 200
Lungs and liver, medullary sarcoma 274
Lupus treated by the iodine ....... 570

M.

M'Fadzen, Mr. on water-dressing.. 551
M'Lellan, Dr. his Parisian formulary 540
Madeira best adapted for phthisis.. 92
Mamma, excision of the.......... 191
Marley, Mr. on diseases of children 553
Marshall, Mr. on vaccination ...... 499
Materialists *versus* immaterialists .. 309
Maxwell, Mr. his marvellous case.. 498
Medical Provident Institution .... 484
Medicine, popular illustrations of .. 289
Medullary sarcoma of the stomach 223
Medullary sarcoma in the ham .... 274
Melted wax for ulcers ............ 37
Mercury, diseased testicle cured by 231
Mercury in ovarian disease......... 429
Microscopical researches in anatomy 51
Military surgery, principles of .... 361
Mills, Dr. his cases of apoplexy.... 204
Milt-like tumour, Dr. Monro on the 265
Mitral valve, true action of the.... 159
Mitral valve, cases of disease of the 555
Modern medical ethics and maxims 145
Monro, Dr. on medullary sarcoma 224
Montgomery, Mr. his ligature ultrà aneurisma ................... 165
Montgomery, Mr. on carotid aneurism ......................... 502
Morphine, M. Bally on .......... 545

N.

Nerve, spiral, tumour of.......... 184
Nerves, excision of .............. 470
Nervous system ................. 22
Neuralgia of the heart............ 122
Neuralgia of the stomach ........ 125
Neuralgia, Dr. Palmer on ........ 310
Neuralgic diseases, Mr. Teale on.. 112
Nitrate of silver in leucorrhœa .... 517
Nitre in scurvy, Mr. Cameron on .. 483
Note takers, rival................ 193
Nothing new under the sun ...... 247
Nux vomica, paraplegia cured by .. 203

O.

Obscure peritonitis, cases of ...... 478
Odontoid process, caries of the .... 489
Oil of turpentine, Dr. Moran on the 211
Oil of turpentine in iritis ........ 232
Ophthalmia, acute purulent ...... 196
Ophthalmia, intermittent ........ 534
Opium in infantile diseases. ...... 553
Osteo-sarcoma, removal of........ 469
Ovaria, structural diseases of the .. 417
Ovaria, cysts of the.............. 419
Ovaria, scirrhus of the............ 421
Ovaria, fungoid disease of the...... 422
Ovaria, cases of extirpation of the.. 440
Ovaria, the seat of the soul........ 462
Ovarian disease, treatment of ..... 426
Oxalic acid, tests for ............ 496

P.

Pain in the side, hysterical........ 292
Palmer, Dr. his popular medicine .. 289
Paracentesis thoracis ............ 468
Paralysis, strychnia in............ 130
Paralysis, ascending, curious case of 168
Paralysis, dorsal vertebræ removed 207
Paraplegia cured by nux vomica ... 203
Parisian hospitals, formulary of the 540
Partial ramollissement of the brain 215
Parturition, affections of the joints 33
Penis, amputation of the.......... 277
Percussion and pressure in ovarian tumours ..................... 431
Pericranium, peculiar affection of the 256

Peritonitis, obscure, cases of ...... 478
Phlebitis, Mr. Arnott on .......... 1
Phlebitis, fatal cases of .......... 5
Phlebitis, secondary affection in.... 19
Phlebitis, uterine, Dr. Lee on .... 376
Phlegmatia dolens, cases of....... 377
Phlegmatia dolens, critical remarks 381
Phrenology, remarks on .......... 291
Phthisis, Dr. Clark on climate in .. 90
Phthisis, causes of .............. 100
Phthisis, symptomatology of ...... 102
Phthisis, percussion and auscultation in ...................... 104
Phthisis, complications of ........ 106
Phthisis, course of .............. 107
Phthisis, treatment of............ 108
Phthisis, climate in.............. 109
Phthisis, is it contagious? ........ 111
Phthisis pulmonalis, Dr. Parrish on 241
Physicians, soirées at the College of 520
Physics, Dr. Arnott's elements of.. 334
Pneumo-thorax, interesting case of 466
Poisons, Dr. Christison's treatise on 362
Poisoning, general theory of ...... 365
Poisoning, symptoms of .......... 369
Poisoning, imaginary or pretended 374
Polypus of the uterus, Dr. Gooch on 321
Polypus uteri, remarkable case of.. 324
Polypus uteri, diagnosis of........ 325
Polypus uteri, ligature for ........ 326
Pompeii, "Weiss's dilator" found in 247
Post-mortem examinations of the kings of France................ 259
Pregnancy, Dr. Gooch on ........ 42
Pregnancy, fallacious symptoms of, 46
Press, radical, liberty of the ...... 438
Prichard, Dr. on the vital principle 316
Procidentia uteri, case of ........ 162
Prolapsus ani.................... 154
Prostate, incision of, in lithotomy.. 73
Psoriasis diffusa, case of .......... 276
Puff, professional................ 146
Pulmonary apoplexy, cases of ..... 87
Pulmonary phthisis, eclectic article 97
Pulmonary apoplexy, curious cases of 555
Purification, of thames water...... 443
Purulent depôts, Mr. Arnott's theory 21
Purulent depôts, curious case of .. 382
Pylorus, scirrhus of the .......... 164

R.

Radius, head of, dislocated backwards 178
Raikem, Dr. on inflammation of the spleen........................ 252
Ramollissement, partial, of the brain 215
Ramollissement of the spinal cord.. 487
Regulations of the Apothecaries' Company .................... 169
Remuneration of general practitioners ...................... 486
Respiratory organs, affection of, in fever ........................ 393
Rhino-plastic operation, case of ... 268
Rival note-takers................ 193

S.

Sane or insane.................. 509
Scalp, diffuse inflammation of the, 172
Scirrhous pylorus, case of ........ 164
Scirrhus of the ovarium .......... 421
Scotland, Medical Provident Institution of ...................... 484
Scott, Mr. on lavements.......... 499
Scrofula, M. Dupuytren's treatment of ...................... 554
Scrotum, elephantiasis of ........ 535
Scrotum, *anomalous* affection of the, 498
Scrotum, morbid itching of the .... 493
Scrotum, elephantiasis of the...... 505
Scurvy, nitrate of potass in ....... 483
Secret, grand, for a successful and honourable career ............ 147
Section, Cæsarian, on a dead woman, 521
Simple and compound fractures.... 566
Skin, anatomy of the ............ 60
Skull, compound fracture of the .. 276
Smith, Dr. on fever.............. 337
Smith, Dr. on fever.............. 385
Solar heat, influence of .......... 335
Soul, seat of the .............. .. 461
Specks on cornea, M. Dupuytren's treatment .................... 539
Spinal marrow, apoplexy of the.... 88
Spinal marrow, irritation of the ... 113
Spinal cord, ramollissement of the, 487
Spleen reduced into a pulpy mass.. 85
Spleen, Dr. Raikem on inflammation of the...................... 252
Splenitis, case of ................ 83
Stafford, Mr. on ulcers .......... 37
Stammering, Dr. Palmer on ...... 304
Stanley, Mr. on lithotomy ........ 69
Stomach, medullary sarcoma of the, 223
Stomach, irritation of the, simulating apoplexy .................. 251
Stomach and liver, fungus hæmatodes of ...................... 563
Stricture, fatal, of the colon ...... 504
Strychnia in paralysis ........... 130
Strychnia in chronic diarrhœa .... 136
Strychnia in amenorrhœa ........ 137
Submaxillary gland, fungus hæmatodes of the .................. 237
Surgeons, Irish College of, curriculum of the.................... 446
Suture, employment of, in vesico-vaginal fistula ................ 437
Syme, Mr. his treatment of ulcers, 474
Sympathetic ganglia, irritation of the ......................... 119

Synochus gravior, description of the, 349
Syphilis, Mr. Bacot on ........... 548

T.

Tapping for ovarian dropsy ....... 427
Teale, Mr. on neuralgic diseases ... 112
Teallier, M. on iliac abscesses and tumours ..................... 160
Temperature, influence of, on decomposition .................. 335
Temperature of different localities, table of the .................. 94
Testicle, diseased, cured by mercury 231
Tests for oxalic acid............ 496
Thames-water, spontaneous purification of ..................... 443
Thompson, Dr. on anatomy ...... 332
Thorax, morbid appearances of, in fever ....................... 357
Thwaites, Dr. his case of fungus hæmatodes ..................... 563
Tic douloureux, excision of nerves in ......................... 470
Tight-lacing, injurious effects of .. 512
Tissues, various, of the human body, 57
Titley, Dr. on elephantiasis of the scrotum ...................... 505
Touch, examination by, in pregnancy 44
Toxicology, Dr. Christison's work on ........................... 362
Trephining, epilepsy cured by .... 504
Tubercular phthisis, eclectic article on ......................... 97
Tuber annulare, extravasation in the ......................... 458
Tumour removed from the spinal nerve ........................ 184
Tumour, milt-like, Dr. Monro on the, 265
Turpentine, oil of, Dr. Moran on the ......................... 211
Tweedie, Dr. on fever........... 337
Tweedie, Dr. on fever ........... 385
Typhus, strong instance of the contagion of .................... 308
Typhus, Dr. Smith on........... 353
Typhus, Dr. Tweedie on.......... 395

U.

Ulcers, Mr. Stafford on .......... 37
Ulcers, Mr. Syme's treatment of .. 474
Ulcers, water dressing in ........ 551
Umbilical hernia, remarkable case of 463
Urine, fatal extravasation of ...... 275
Urinary Calculi, Dr. England on .. 481
Uterine phlebitis, Dr. Lee on .... 376
Uterus, Dr. Gooch on polypus of the 321
Uterus, hydatids in the .......... 47
Uterus, prolapsus of, case of...... 162
Uterus, peculiar hæmorrhage from the ......................... 194
Uwins, Dr. David, letter to ...... 278

V.

Vaccination, popular summary of.. 499
Vaccination of the fœtus in utero.. 214
Vagina, artificial anus opening into the ......................... 151
Veins, cases of inflammation of the 377
Veins, Mr. Arnott on inflammation of the ....................... 1
Venæsection, aneurism after ...... 476
Vesico-vaginal fistula, M. Dupuytren on ...................... 271
Vesico-vaginal fistula, Mr. Earle on 272
Vesico-vaginal fistula, suture for .. 437
Viper-bite, severe effects from .... 175
Viscera, affections of, after injuries 24
Viscera, affections of, after injuries of the head.................. 28
Viscera, affections of, after parturition......................... 31
Visceral deposites, independent of phlebitis..................... 26
Vital principle, Dr. Prichard on the 316

W.

Water-dressing, Mr. M'Fadzen on.. 551
Weir, Dr. on delirium tremens .... 568
Williams, Dr. on the auscultation of the heart .................... 155
Wounds in dissection, Mr. Lawrence on .......................... 529

